# Body Position Modulates Gastric Emptying and Affects the Post-Prandial Rise in Plasma Amino Acid Concentrations Following Protein Ingestion in Humans

**DOI:** 10.3390/nu8040221

**Published:** 2016-04-13

**Authors:** Andrew M. Holwerda, Kaatje Lenaerts, Jörgen Bierau, Luc J. C. van Loon

**Affiliations:** 1 Department of Human Movement Sciences, NUTRIM School of Nutrition and Translational Research in Metabolism, Maastricht University Medical Centre+, P.O. Box 616, Maastricht 6200 MD, The Netherlands; andy.holwerda@maastrichtuniversity.nl; 2 Department of Surgery, NUTRIM School of Nutrition and Translational Research in Metabolism, Maastricht University Medical Centre+, P.O. Box 616, Maastricht 6200 MD, The Netherlands; kaatje.lenaerts@maastrichtuniversity.nl; 3 Department of Clinical Genetics, Maastricht University Medical Centre+, P.O. Box 616, Maastricht 6200 MD, The Netherlands; jorgen.bierau@mumc.nl

**Keywords:** protein, body position, digestion, gastric emptying

## Abstract

Dietary protein digestion and amino acid absorption kinetics determine the post-prandial muscle protein synthetic response. Body position may affect gastrointestinal function and modulate the post-prandial rise in plasma amino acid availability. We aimed to assess the impact of body position on gastric emptying rate and the post-prandial rise in plasma amino acid concentrations following ingestion of a single, meal-like amount of protein. In a randomized, cross-over design, eight healthy males (25 ± 2 years, 23.9 ± 0.8 kg·m^−2^) ingested 22 g protein and 1.5 g paracetamol (acetaminophen) in an upright seated position (control) and in a −20° head-down tilted position (inversion). Blood samples were collected during a 240-min post-prandial period and analyzed for paracetamol and plasma amino acid concentrations to assess gastric emptying rate and post-prandial amino acid availability, respectively. Peak plasma leucine concentrations were lower in the inversion compared with the control treatment (177 ± 15 *vs*. 236 ± 15 mmol·L^−1^, *p* < 0.05), which was accompanied by a lower plasma essential amino acid (EAA) response over 240 min (31,956 ± 6441 *vs*. 50,351 ± 4015 AU; *p* < 0.05). Peak plasma paracetamol concentrations were lower in the inversion *vs*. control treatment (5.8 ± 1.1 *vs*. 10.0 ± 0.6 mg·L^−1^, *p* < 0.05). Gastric emptying rate and post-prandial plasma amino acid availability are significantly decreased after protein ingestion in a head-down tilted position. Therefore, upright body positioning should be considered when aiming to augment post-prandial muscle protein accretion in both health and disease.

## 1. Introduction

Ingestion of dietary protein increases muscle protein synthesis rates [1,2,3,4,5]. This increase in muscle protein synthesis has been attributed to the post-prandial rise in circulating (essential) amino acid concentrations, with leucine being of particular relevance [6,7,8,9]. The post-prandial muscle protein synthetic response is regulated on multiple levels, ranging from protein digestion and amino acid absorption [10,11], post-prandial insulin release, and the subsequent increase in muscle perfusion [12], amino acid uptake in muscle [13], activation of anabolic signaling pathways [9,14,15], and subsequent myofibrillar protein synthesis [1,2,3,4,5].

The post-prandial muscle protein synthetic response can be modulated by changing the type [10,16], amount [3,4,17,18], and timing [19,20] of protein ingestion. Differences in the anabolic properties of various proteins have been attributed to their specific protein digestion and amino acid absorption kinetics [21], as well as their amino acid composition [22]. Previous work from our group [16] as well as others [3,23,24,25,26,27,28,29] has shown that the ingestion of more rapidly digestible protein, such as whey, results in a greater post-prandial rise in circulating amino acid concentrations, thereby further increasing whole body and/or muscle protein synthesis rates when compared with the ingestion of a more slowly digestible protein, such as intact casein. However, whey and casein do not only differ in their protein digestion and absorption kinetics, but also in their amino acid composition, with whey containing more leucine [11,24,27].

To define the impact of dietary protein digestion and absorption kinetics *per se* on post-prandial protein handling, we assessed protein digestion and amino acid absorption together with post-prandial muscle protein synthesis rates following the ingestion of intact micellar casein in comparison with its hydrolyzed form [10,16]. Ingestion of the more rapidly digestible casein hydrolysate resulted in a more rapid post-prandial rise in circulating amino acids, greater post-prandial dietary protein-derived amino acid availability, and ~30% higher post-prandial muscle protein synthesis rates when compared to ingestion of intact casein [10]. These findings confirmed the importance of the rate of digestion and amino acid absorption in stimulating post-prandial muscle protein synthesis rates. These findings sparked research investigating various factors that may modulate post-prandial protein handling, which include the type of protein [16,26,27], the matrix in which the protein is consumed [30,31,32], the macronutrient composition of a protein-rich meal [33,34], as well as food preparation [35,36,37] and mastication [38]. We speculated that protein digestion and absorption might also be modulated by a factor as simple as body position (*i.e.*, sitting upright or lying down). Digestive modulation through changes in body position may be of particular interest since a blunted muscle protein synthesis response to feeding is typically observed in settings where patients are subjected to prolonged supine rest or in the absence of gravity [39].

In the present study, we tested our hypothesis that changes in body position strongly modulate gastric emptying rate and affect the post-prandial rise in amino acid concentrations *in vivo* in humans. To test this hypothesis, eight young males were selected to participate in this randomized, cross-over study with two treatments. We assessed gastric emptying rate and the post-prandial rise in circulating amino acids following ingestion of a single, meal-like amount of protein while maintaining a conventional, upright seated body position and while laying supine at a −20° head-down tilted position. This study is the first proof-of-principle study to show that gastric emptying rate is reduced and post-prandial plasma amino acid responses are attenuated when food is ingested in a partly inverted *versus* upright body position.

## 2. Materials and Methods

### 2.1. Subjects

A total of 8 healthy young males (25 ± 2 years, 23.9 ± 0.8 kg·m^−2^) were recruited to participate in the study. All subjects were instructed to refrain from any exhaustive physical activity and alcohol consumption for two days prior to each trial. Subjects remained fasted from 2200 h in the evening before each test. This study is part of a larger project (Netherlands Trial Register, NTR5027) that was approved by the Medical Ethical Committee of the Maastricht University Medical Centre+, The Netherlands (METC143052) and conformed to standards for the use of human subjects in research as outlined in the seventh revision of the Declaration of Helsinki (Brazil, 2013).

### 2.2. Experimental Protocol

This randomized, cross-over study compared the effect of body position on gastric emptying rate and plasma amino acid availability after protein ingestion. In a proof-of-principle approach, we chose to assess potential differences in body position by comparing an upright seated body position with a moderately inverted body position. Therefore, subjects consumed the protein bolus in an upright seated position (control) and in a −20° head-down tilted position (inversion). On the test days, participants reported to the lab in a fasted state and had one Teflon catheter inserted into the antecubital vein of the right arm. A background blood sample was collected, and blood pressure and heart rate were measured before subjects were positioned in either the control or inversion position, completed in a randomized order. For the control treatment, subjects sat in a standardized, stationary chair and were instructed to hold their lower back against the back support of the chair. Subjects were supervised throughout the test day and were constantly reminded to maintain the upright posture. For the inversion treatment, subjects were secured with shoulder and waist straps onto a rotatable bed (Confidence Inversion Table, Confidence Fitness, UK) for the duration of the test. Once secured, the table was tilted to an angle of −20° head-down from a horizontal position, confirmed using an electronic level. Subjects acclimatized to each body position for 30 min after which a baseline blood sample was drawn, and blood pressure and heart rate were measured. Following the baseline blood draw, subjects ingested 60 g of skim milk powder (Campina, The Netherlands—Energy (En): 890 kJ, Protein: 22 g, Carbohydrate: 29 g; Fat: 0.4 g) plus 1.5 g paracetamol (acetaminophen, Kruidvat, Leiden, The Netherlands) dissolved in water up to a volume of 500 mL. Paracetamol was added to the beverage to assess gastric emptying rate. The protein bolus was ingested within a 5-min time period for both treatments. Following ingestion of the drink (*t* = 0 min), blood samples were collected at *t* = 15, 30, 45, 60, 90, 120, 180, 240 min. After the last blood sample was collected, subjects were slowly returned to an upright body position and re-acclimatized throughout a 10-min time period.

### 2.3. Gastric Emptying

Gastric emptying rate was assessed by measuring the post-prandial rise in plasma paracetamol (acetaminophen) concentrations after ingestion of protein with 1.5 g of paracetamol added [40,41]. With paracetamol being rapidly absorbed in the small intestine, gastric emptying forms the rate-limiting step determining the appearance rate of paracetamol in the circulation. Peak plasma paracetamol concentrations are typically reached after 30–60 min following ingestion with a *t*1/2 of ~2 h [42]. We used plasma paracetamol appearance in the circulation as a marker of gastric emptying rate as applied previously in our laboratory [42] as well as others [43,44,45].

### 2.4. Plasma Analysis

Blood samples were collected in tubes containing EDTA and centrifuged at 1000× *g* for 10 min at 4 °C. Aliquots of plasma were frozen in liquid nitrogen and stored at −80 °C until further analyses. Plasma glucose and insulin concentrations were analyzed using commercially available kits (Glucose HK CP, Horiba ABX Diagnostics, France, Ref: AA11A01667, and Human Insulin-specific (RIA), Merck Millipore, Germany, Cat #: HI-14K, respectively). Plasma (100 µL) for amino acid analyses was deproteinized on ice with 5-sulphosalicylic acid and mixed, and the clear supernatant was collected after centrifugation. Amino acid profiles were determined using ultra-performance liquid chromatography tandem mass spectrometry (UPLC-MS/MS) as described previously [46]. Plasma paracetamol concentrations were analyzed with an acetaminophen assay kit (K991598, Roche, Basel, Switzerland) with the COBAS-Integra 800 immuno-assay analyzer. Briefly, acetaminophen is hydrolyzed to *p*-aminophenol and acetate. The *p*-aminophenol is then converted to an indophenol by enzymatic reaction. The production of indophenol is colorimetrically analyzed and is directly proportional to acetaminophen concentration in plasma.

### 2.5. Statistics

All data are displayed as means + standard error of means (SEM). Time-dependent variables such as plasma glucose, insulin, amino acid, and paracetamol concentrations were analyzed using a two-way repeated measure ANOVA (time × treatment). Upon identification of a significant time × treatment interaction, Bonferroni *post hoc* testing was used to identify time points at which the treatments differed. Differences in treatment-dependent variables, such as area under the curve (AUC), were analyzed using a Student’s paired *t*-test. Pearson’s *r* product moment correlation was used to examine the linear relationship between plasma essential amino acids (EAAs) and paracetamol AUC for each test. Statistical significance was set at *p* < 0.05. All calculations were performed using SPSS 21.0 (SPSS Inc., Chicago, IL, USA).

## 3. Results

### 3.1. Plasma Glucose and Insulin Concentrations

**Figure 1:**
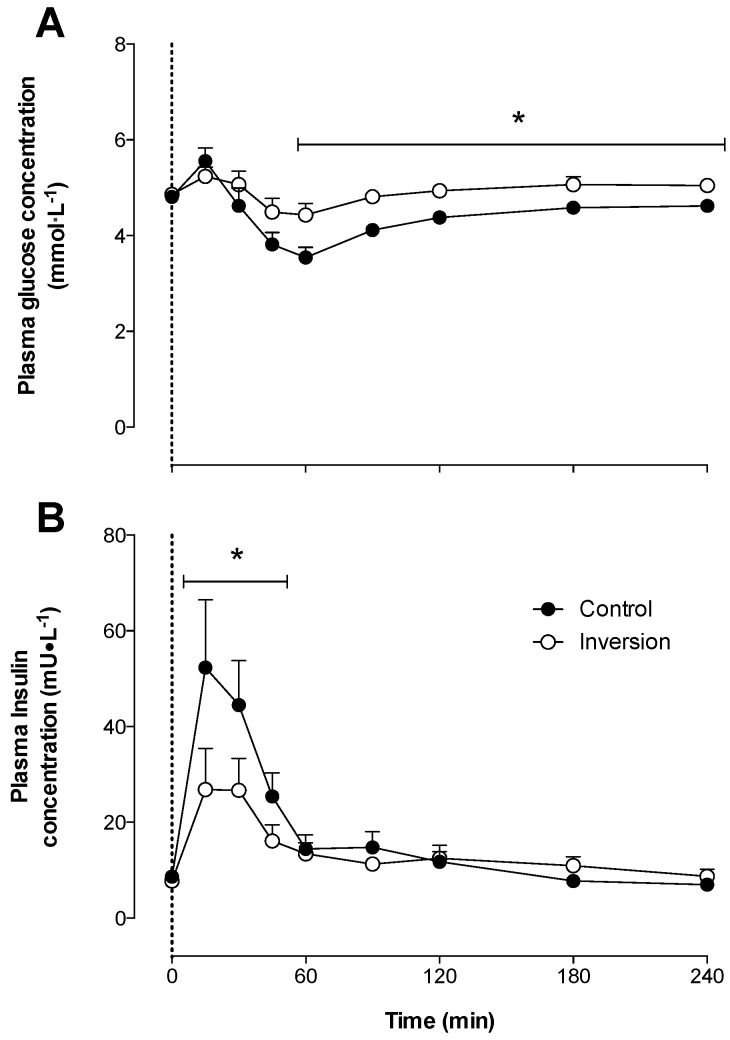
Mean (+SEM) plasma glucose (mmol·L^−1^) (**A**) and insulin (mU·L^−1^) (**B**) concentrations after protein ingestion while seated (Control; *n* = 8) and in a −20° head-down tilted position (Inversion; *n* = 8). The data were analyzed with a two-way repeated-measures (treatment × time) ANOVA. Glucose: time effect: *p* < 0.05; time × treatment interaction: *p* < 0.05. Insulin: time effect: *p* < 0.05; time × treatment interaction: *p* < 0.05. * Significant differences (*p* < 0.05) between treatments within each time point.

Plasma glucose (Figure 1A) and insulin (Figure 1B) concentrations increased following protein ingestion in both treatments (*p* < 0.05). After reaching peak concentrations, plasma glucose concentrations decreased in both treatments and were significantly lower in the control *vs*. inversion treatment between *t* = 60–240 min (time × treatment interaction, *p* < 0.05). The increase in insulin was significantly different between the inversion and control treatments (time × treatment interaction, *p* < 0.05). Peak post-prandial insulin concentrations were reached at *t* = 15 min and were significantly lower in the inversion *vs*. control treatment (27 ± 8 *vs*. 52 ± 13 mU·L^−1^, *p* < 0.05).

### 3.2. Plasma Amino Acid Responses

**Figure 2:**
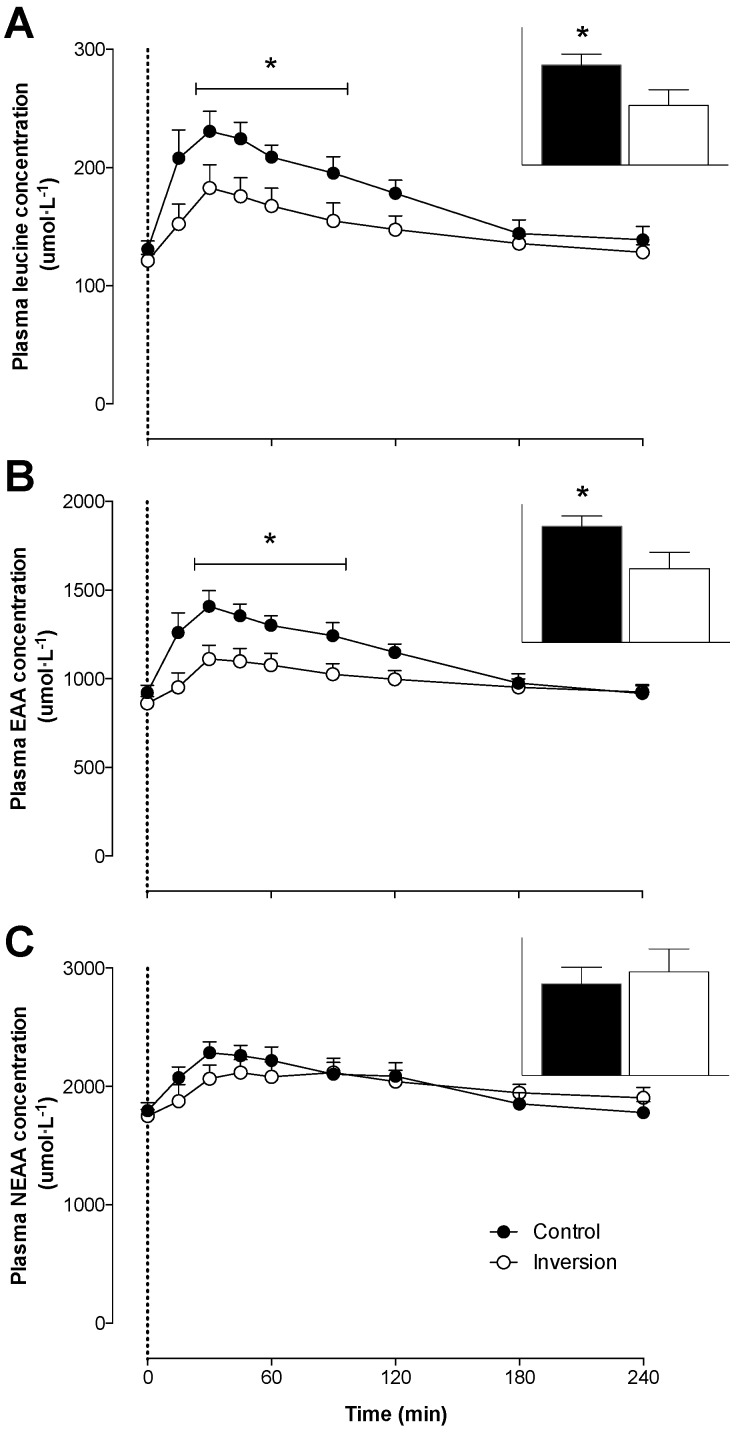
Mean (+SEM) plasma leucine (**A**), essential (EAA) (**B**), and non-essential (NEAAs) (**C**) amino acid concentrations (umol·L^−1^) after protein ingestion in a seated (Control; *n* = 8) and in a −20° head-down tilted position (Inversion; *n* = 8). The data were analyzed with a two-way repeated-measures (treatment × time) ANOVA. Leucine: time effect: *p* < 0.01; time × treatment interaction: *p* < 0.05. EAAs: time effect: *p* < 0.01; time × treatment interaction: *p* < 0.05. NEAAs: time effect: *p* < 0.01; time × treatment interaction: *p* > 0.05. Area under the curve over 240 min (arbitrary units, AU) inset and analyzed with a Student’s paired *t*-test. * Significant differences (*p* < 0.05) between treatments within each time point.

Plasma leucine (Figure 2A) and EAA (Figure 2B) concentrations increased following protein ingestion in both treatments (*p* < 0.01). Plasma leucine and total EAA concentrations showed an attenuated rise in the inversion compared with the control treatment, with concentrations being significantly lower in the inversion treatment between *t* = 15–120 min (*p* < 0.05). Overall leucine and EAA availability, expressed as AUC calculated over 240 min, were lower in the inversion compared with control treatment (*p* < 0.05). No significant differences were observed in post-prandial non-essential amino acid (NEAA) availability (Figure 2C) between treatments (main effect for time, *p* < 0.05; time × treatment interaction, *p* > 0.05).

### 3.3. Gastric Emptying

**Figure 3:**
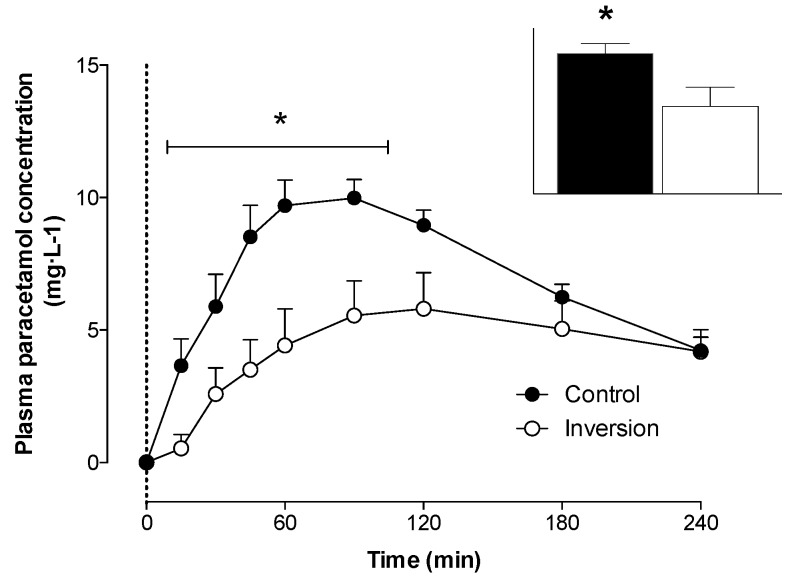
Mean (+SEM) plasma paracetamol concentrations (mg·L^−1^) after protein ingestion in a seated (Control; *n* = 7) and a −20° head-down tilted position (Inversion; *n* = 7). The data were analyzed with a two-way repeated-measures (treatment × time) ANOVA. Time effect: *p* < 0.05; time × treatment interaction: *p* < 0.05. Area under the curve over 240 min (arbitrary units, AU) inset and analyzed with a Student’s paired *t*-test. * Significant differences (*p* < 0.05) between treatments within each time point.

Plasma paracetamol (Figure 3) concentrations increased following protein ingestion in both treatments (*p* < 0.05). An attenuated post-prandial rise in paracetamol concentrations was observed in the inversion compared with control treatment (time × treatment, *p* < 0.05). Peak paracetamol concentrations reached 10.0 ± 0.6 mg·L^−1^ at *t* = 90 min in the control treatment and 5.8 ± 1.1 mg·L^−1^ at *t* = 120 min (*p* < 0.05) in the inversion treatment. Gastric emptying rate during the early post-prandial phase, assessed by paracetamol AUC from *t* = 0–60 min was 54% ± 13% lower in the inversion compared with control treatment (132 ± 37 *vs*. 344 ± 47 AU (arbitrary units); *p* < 0.05). When assessed over the entire 240-min post-prandial period, paracetamol AUC was 38% ± 13% lower in the inversion compared with control treatment (1054 ± 196 *vs*. 1693 ± 101 AU; *p* < 0.05).

**Figure 4:**
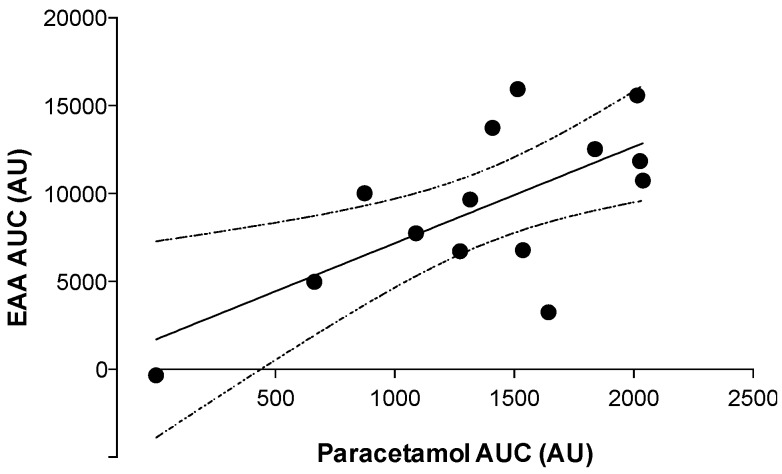
Correlation between post-prandial plasma EAA and paracetamol availability. Values represent area under the curve (AUC) calculations based on plasma EAA and paracetamol concentrations measured over 240 min in each trial (*n* = 14, one subject on both trials did not ingest paracetamol). The solid line indicates the linear regression line of best fit, and the dashed lines represent the 95% confidence interval. A significant positive correlation was observed (*r* = 0.68; *p* < 0.01).

Pearson’s *r* product moment correlation was performed between plasma EAAs and paracetamol AUCs over the 240-min post-prandial period (Figure 4). A significant positive (*r* = 0.68; *p* < 0.01) correlation was detected between plasma EAAs and paracetamol AUCs.

## 4. Discussion

In the present study, we observed that gastric emptying rates were reduced when protein was ingested in an inverted *versus* upright seated position. The post-prandial rise in circulating amino acid concentrations was attenuated following protein ingestion in the head-down tilted position along with substantially lower post-prandial amino acid availability when compared to feeding in the upright seated position.

It has been well established that the post-prandial rise in plasma EAA concentrations following food ingestion increases muscle protein synthesis rates [6,7,8,47]. In the present study, we observed a rapid rise in plasma glucose and insulin (Figure 1) and EAA (Figure 2) concentrations following ingestion of a single, meal-like amount of protein when sitting in a normal, upright position. Total EAA concentrations increased rapidly, reaching peak levels of 1408 ± 80 umol·L^−1^ within 30 min after ingesting 22 g of milk protein. In line, plasma leucine concentrations rapidly reached peak levels of 236 ± 15 umol·L^−1^ within the same post-prandial time frame (Figure 2A). The level of increase in plasma EAA concentrations, and leucine in particular, observed in the control treatment have been previously shown to result in post-prandial muscle protein synthesis rates that were 30%–100% higher when compared to basal, post-absorptive muscle protein synthesis rates [3,4,5,30,33,48].

When the same bolus of protein was ingested in an inverted body position, the post-prandial rises in plasma insulin (Figure 1B), EAA (Figure 2B), and leucine (Figure 2A) concentrations were substantially reduced. Peak plasma EAAs, and leucine in particular, reached levels of 1112 ± 70 and 177 ± 15 umol·L^−1^, respectively, which were substantially lower when compared to levels observed after ingesting protein in an upright seated position (*p* < 0.05). The extent of the difference in the post-prandial rise in amino acid concentrations following ingestion of a meal-like amount of protein in an inverted *versus* a normal, upright seated position was beyond our expectations. The blunted post-prandial rise in plasma EAA concentrations, and leucine concentrations in particular, observed in the inversion treatment would unlikely suffice to induce a measurable increase in post-prandial muscle protein synthesis rates as shown in previous work [4,18].

Besides the blunted post-prandial rise in amino acid concentrations following feeding in the inverted *versus* upright seated position, we also observed lower plasma EAA and leucine availability over the 4-h post-prandial period (Figure 2). These findings are in line with Koopman *et al*., who showed that ingestion of a single 35 g bolus of intact, micellar casein resulted in less protein-derived amino acids being released into the circulation when compared with the ingestion of the same amount of hydrolyzed casein [10]. The reduction in protein-derived amino acid release could be attributed to reduced gastric emptying rates and/or greater splanchnic amino acid retention following ingestion of a more slowly digested protein. In the present study, we assessed gastric emptying rates by measuring the increase in plasma paracetamol concentrations following the co-ingestion of 1.5 g paracetamol [40,41,42,49]. Peak plasma paracetamol concentration was substantially lower (5.8 *vs*. 10.0 mg·L^−1^) and was reached 30 min later when protein was ingested in the inverted compared with the upright seated position (Figure 3). The attenuated rise in plasma paracetamol concentrations following protein ingestion implies that gastric emptying rates were substantially reduced in the inverted *versus* upright seated position. Furthermore, we found that the overall appearance of plasma paracetamol was positively correlated with the overall plasma EAA availability (Figure 4). The reduction in gastric emptying rate demonstrated in the inverted position may be partly explained by recently published work using computer modeling techniques to demonstrate that the movement of stomach content is slowed in a supine position due to a lower amount of stomach content gathering near the pylorus [50]. Therefore, we conclude that the attenuated post-prandial rise in plasma amino acid concentrations following ingestion of protein in an inverted compared with an upright seated position can, at least partly, be attributed to a reduction in gastric emptying rate.

The present findings may be of relevance in various settings of disuse atrophy. Disuse atrophy has been partly attributed to a reduced sensitivity of the muscle protein synthetic response to the post-prandial rise in plasma amino acid concentrations, and leucine concentrations in particular [51,52]. Feeding hospitalized patients in a supine as opposed to a normal upright sitting position may attenuate the post-prandial rise in plasma amino acid concentrations, contributing to anabolic resistance in these patients. Proper upright body positioning may be an important pre-requisite to increase the post-prandial rise in plasma amino acid availability, thereby increasing the post-prandial muscle protein synthetic response to meal ingestion. In other words, our mothers may have been right in more than one way by telling us to sit up straight at the dinner table. Lastly, the present findings imply that part of the muscle lost by astronauts due to microgravity [53,54] may also be explained by reduced protein digestion and amino acid absorption, thereby attenuating the post-prandial rise in plasma amino acid concentrations and lowering the post-prandial muscle protein synthetic response to food intake.

## 5. Conclusions

Changes in body position substantially modulate gastric emptying rate and the subsequent post-prandial rise in plasma amino acid availability. Therefore, an upright body position during and after feeding is important for adequate nutrient absorption and should be considered when aiming to optimize post-prandial muscle protein accretion in both health and disease.

## Abbreviations

The following abbreviations are used in this manuscript:

AU	Arbitrary unit
AUC	Area under the curve
EAA	Essential amino acid
NEAA	Non-essential amino acid
SEM	Standard error of the mean
